# New brain lesions with no impact on physical disability can impact cognition in early multiple sclerosis: A ten-year longitudinal study

**DOI:** 10.1371/journal.pone.0184650

**Published:** 2017-11-17

**Authors:** D. Wybrecht, F. Reuter, F. Pariollaud, W. Zaaraoui, A. Le Troter, A. Rico, S. Confort-Gouny, E. Soulier, M. Guye, A. Maarouf, J-P. Ranjeva, J. Pelletier, B. Audoin

**Affiliations:** 1 Aix-Marseille Université, CNRS, CRMBM UMR 7339, Marseille, France; 2 Hôpital d’Instruction des Armées Sainte Anne, Toulon, France; 3 APHM, Hôpital de la Timone, Pôle de Neurosciences Cliniques, Service de Neurologie, Marseille, France; 4 APHM, Hôpital de la Timone, Pôle d’Imagerie Médicale, CEMEREM, Marseille, France; Washington University, UNITED STATES

## Abstract

**Objective:**

In early multiple sclerosis, although brain T2 lesions accrual are hallmark of the disease, only weak correlations were found between T2 lesions accrual and EDSS progression, the disability scale commonly used in multiple sclerosis studies. This may be related to the very poor sensitivity of EDSS to cognitive dysfunctions that may occur and progress from the first stage of the disease. In the present study, we aimed to demonstrate that cognitive deficits progress during the first ten years of MS and are significantly impacted by new T2 lesions.

**Methods:**

EDSS and extensive neuropsychological battery (22 measures) exploring memory, attention/speed of information processing and executive functions were assessed at baseline, Year 1 and Year 10 in 26 patients enrolled after their first clinical attack. To limit the bias of test-retest effect, only measures obtained at Year 1 and Year 10 were reported in the analysis. Raw scores of patients were transformed into z-scores using published normative data when available or scores of matched controls. Lesion probability mapping was used to assess the potential relationships between T2 lesions accumulation, cognitive decline and EDSS progression (P<0.05, FWE-corrected).

**Results:**

At Year 1, 27% of patients showed attention/speed of information processing deficits, 11.5% executive dysfunction and 11.5% memory impairment. During the follow-up, frequency and severity of executive dysfunction increased (from 11.5% of patients at Year 1 to 42% at Year 10, p<0.01) while no significant changes were evidenced for the other cognitive domains. Median EDSS increased from 0.5 [range: 0–3] at Year 1 to 2.5 [range: 0–6.5] at Year 10 (p<0.001). During the ten-year follow-up, lesions accumulation in the left cerebellum and semi-ovale centers was associated with EDSS progression. In contrast, most lesions accumulation in the frontal, parietal and temporal lobes were associated with cognitive decline but had no effect on EDSS progression.

**Conclusion:**

The present study provides strong evidence that clinically silent T_2_ lesions impact cognition in early MS. In daily practice, early prevention of T2 lesions accrual may be useful to limit cognitive decline.

## Introduction

Multiple sclerosis (MS) is the first non-traumatic cause of disability in young people, responsible for physical but also cognitive deficits. Contribution of brain T2 lesions to irreversible disability has been generally interpreted as modest considering the weakness of the correlations found between T2 lesions load and disability [1, 2]. However, disability was usually measured by Expanded Disability Status Scale (EDSS) that is mainly sensitive to physical deficits. Lesions accumulation in the brain may be more associated with cognitive deficit [3–5]. It is now established that cognitive deficits are common in MS, affecting from 40 to 70% of patients [6, 7]. Cognitive impairment is present from the first clinical event of the disease affecting mainly memory, attention/speed of information processing and executive functions [7–12]. Little is known about the evolution of cognitive impairment during the first stage of the disease and its relationship with lesions accumulation according to the scarcity of longitudinal studies performed, especially at the early stage of the disease. Only few studies have assessed the short-term evolution of cognitive impairment after the first clinical event of MS [9,13–15]. Up to now, no study has assessed middle-term evolution of cognitive impairment in patients included after their first clinical event of MS.

We aimed to demonstrate in the present study that cognitive impairment increases during the first ten years of Relapsing Remitting MS (RRMS) and is highly impacted by brain T2 lesions accumulation. To test this hypothesis, we performed a longitudinal study in patients enrolled after their first clinical event and explored them using extensive neuropsychological battery and conventional Magnetic Resonance Imaging (MRI).

## Patients and methods

### Subjects

Thirty-eight patients were enrolled from june 2000 to may 2004 after a first clinical event highly suggestive of multiple sclerosis in a longitudinal MRI and neuropsychological study. All patients were recruited at the department of Neurology (Timone University Hospital, Marseille) and fulfilled the following criteria: (1) age between 18 and 45 years, (2) occurrence of an inflammatory demyelinating event of the central nervous system, (3) presence of one or more lesion located in the brain at the initial MRI and (4) presence of oligoclonal band in the CSF.

Physical disability was assessed using the Kurtzke Expanded Disability Status Scale [16] at baseline, Year 1 and Year 10.

In a second time, two groups of healthy controls matched for age and educational level according to patients’ characteristics at Year 1 and Year 10 were included (35 controls for Year 1 and 31 for Year 10). Controls were recruited from June 2000 to January 2008 in Marseille.

All participants gave their written informed consent to participating in this study that was approved by the local Ethics Committee (Timone hospital).

### Neuropsychological assessment

Neuropsychological assessment was performed at three time points (baseline, Year 1 and Year 10) in patients. To limit the practice effect, we only included in the analysis data obtained at Year 1 and Year 10.

The different neuropsychological tests have been grouped into cognitive domains as follows:

- *Memory*: Selective Reminding Test, 10/36 Spatial Recall Test for the Brief Repeatable Battery (BRB), free and delayed recalls of the Grober and Buschke test, forward digit and block spans, histories and pictures immediate and delayed recalls from the Weschler Memory Scale-Revised;
- *Attention/ Speed of information processing*: Trail Making Test A, 100-item version of the Stroop (reading words and naming colors), 3s- Paced Auditory Serial Addition Test, Symbol Digit Modalities Test;
- *Executive functions*: naming task with interference of the Stroop test, Trail Making Test B, backward digit and block spans, World List Generation with phonemic (letter P) and semantic (animals) verbal fluencies;

Raw scores of patients for each test were transformed into z-scores using published normative data when available in French (for Grober and Buschke test [17], digit and block spans [18], Weschler Memory Scale-Revised [18], Trail Making Test A and B [19]) or using the raw scores of the matched healthy controls (35 controls for Year 1 and 31 for Year 10). For each cognitive domain the mean Z-score of each patient was calculated. Patients were considered as cognitively impaired in one cognitive domain if the mean of the Z-scores of all the tests composing this domain was below -1.5 [9].

### Imaging

Patients were explored by MRI at Year 1 and Year 10 corresponding to the day of the neuropsychological evaluation. MRI at Year 1 was performed on a 1.5 Tesla commercially available MRI system (Magnetom Vision Plus, Siemens, Erlangen, Germany). MRI at Year 10 was performed on another 1.5 Tesla commercially available MRI system (Magnetom Avanto, Siemens, Erlangen, Germany). MRI protocols were identical and included a transverse fast spin-echo proton density-weighted and T_2_-weighted sequence (2600/15/85 ms [TR/TE1/TE2], 44 contiguous sections, 3-mm section thickness, 256-mm FOV, 256 x 256 matrix, in plane resolution 1mmx1mm).

### Lesion probability mapping

T_2_ lesions were outlined by the same experienced neurologist (DW) onto the T_2_-weighted images by means of a semi automated method written on the interactive data language (IDL platform, Research System, Inc.) in order to assess T_2_ lesions load and to obtain lesions masks for the lesion probability mapping. Original T_2_ scans were normalized to the MNI template space using SPM8 software (Wellcome Department of Cognitive Neurology, University College of London, London, UK) and the parameters of the normalization were applied to the lesions masks. We performed a substraction between T2 normalized lesion masks at Year 1 and at Year 10 to obtain a mask corresponding to the new T2 lesions and enlarged lesions over the follow-up. Finally, these masks were smoothed using a 12-mm FWHM Gaussian kernel. To correlate the lesions probability maps and the clinical data, we applied a voxel-by-voxel multiple linear regression model, using SPM8 software (P<0.05, FWE corrected, K = 20).

### Statistical analysis

All statistical analyses were performed using JMP 9.0.0, SAS Institute Inc. Potential changes of the percentages of patients suffering from cognitive deficits between Year 1 and Year 10 were tested using the Fischer’s exact test. Potential changes of the z-scores of each cognitive domains or EDSS between Year 1 and Year 10 were performed using the Wilcoxon signed-rank test. Potential correlations between T2 lesion load changes, EDSS changes and significant z-scores changes of the cognitive domains between Year 1 and Year 10 were assessed using the Spearman Rank correlation test.

## Results

### Population

Twenty-six patients completed all evaluations of the follow up. Ten patients were lost to follow up, and 2 patients did not perform MRI and neuropsychological tests at Year 1. Table 1 shows demographic, clinical and imaging data of the 26 patients that completed the follow-up. Among them, 22 were women (84%). Mean age of the patients was 32 years, ranging from 19 to 45 years. Median EDSS was 0.5 at Year 1 [range: 0–3] and 2.5 [range: 0–6.5] at Year 10 (p<0.001). At the end of the study, all patients fulfilled the McDonald revised criteria for MS [20]. Fifteen out of 26 patients (57%) received disease-modifying therapies at any time during the follow-up.

**Table 1:** Demographic and clinical data of patients.

		Patients (26)
**Mean age, years (range)**		32 (19–45)
**Sex ratio (W(%)/M)**		22(84%)/4
**Educational level (years)**		11.5 (8–17)
**Nature of CIS (%)**	Optic neuritis	11 (42)
	Myelitis	6 (23)
	Brainstem	3 (11)
	Cerebral hemispheres	3 (11)
	Multifocal	3 (11)
**Delay between symptoms onset and first MRI (months)**		7.6 (0.2–19)
**Delay between baseline and year 1 explorations (years)**		1.04 (0.91–1.42)
**Delay between baseline and year 10 (years)**		10.29 (9.2–13.3)
**Median EDSS at Year 1**		0.5 (0–3)
**Median EDSS at Year 10**		2.5 (0–6.5)

The age and educational level were not different between patients and healthy controls at Year 1 (mean age for the patients 32 ± 6.6 years, for the controls 34.5 ± 6.6 years; p = 0.14; years of education for the patients 11.5±3, for the controls 12±2; p = 0.2), and Year 10 (mean age for the patients 41.5±7 years, for the controls 39±8; p = 0.15; years of education for the patients 11.5±3, for the controls 12.5±2.4; p = 0.1).

### Evolution of cognitive functions in patients

**Fig 1:**
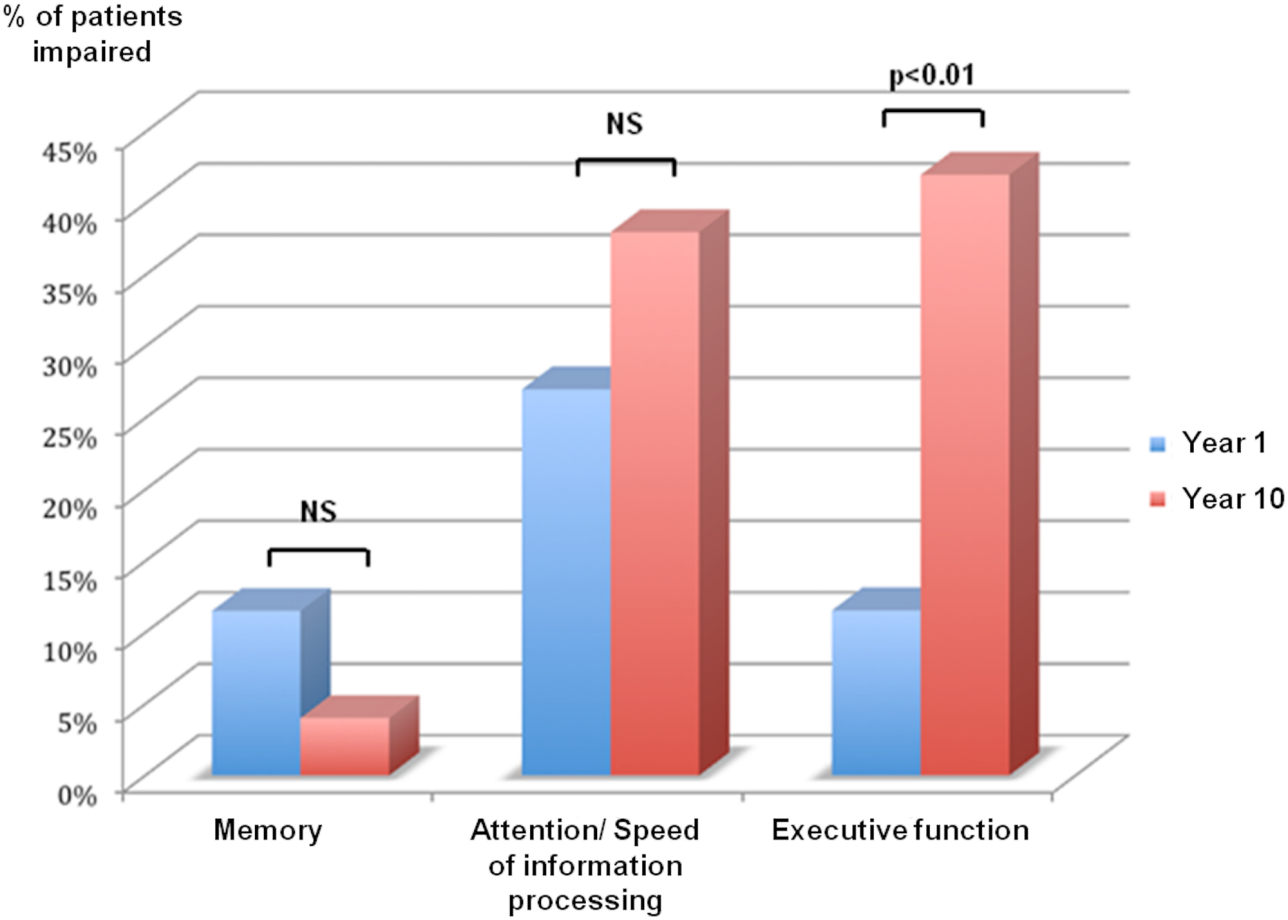
Percentage of patients with Z score below -1.5 for each cognitive domain, at Year 1 and Year 10.

For executive functions, 11.5% of patients were considered as cognitively impaired at Year 1, 42% at Year 10 (p<0.01, Fischer’s exact test). For memory, 11.5% were classified as cognitively impaired at Year 1 and 4% at Year 10 (p = 0.6, Fischer’s exact test). Considering attention and speed of information processing, 27% of patients were classified as impaired at Year 1 and 38% at Year 10 (p = 0.5, Fischer’s exact test). (Fig 1)

50% of patients showed at least one cognitive domain impaired at Year 1 and 54% at Year 10 (p = 0.5, Fischer’s exact test). 19% showed at least two domains impaired at Year 1 and 39% at Year 10 (p = 0.05, Fischer’s exact test). 8% of patients showed at least three cognitive domains impaired at Year 1 and 4% at Year 10 (p = 0.6, Fischer’s exact test).

Between Year 1 and Year 10, patients showed significant decline of executive functions (mean z-score -0.7 (SD = 0.6) at Year 1, -1.13 (SD = 1.15) at Year 10; p = 0.01, Wilcoxon signed-rank test) but no significant changes of memory (mean z scores at Year 1, -0.1 (SD = 0.86), mean z scores at Year 10, -0.4 (SD = 0.6), p = 0.99, Wilcoxon signed-rank test) and attention/speed of information processing (mean z scores at Year 1, -0.8 (SD = 0.96), mean z score at Year 10, -0.65 (SD = 1.16), p = 0.79, Wilcoxon signed-rank test).

Exploring the executive functions which is the only cognitive domain significantly evolving during the ten-year follow-up, we assessed the respective evolution of each test composing this domain. The mean z-scores of the Trail Making Test B significantly decreased from -1.6 (SD = 1.6) at Year 1 to -3.5 (SD = 4.5) at Year 10 (p = 0.04, Wilcoxon signed-rank test). The mean z-scores of the Backward digit span significantly decreased from -0.35 (SD = 0.7) at Year 1 to -0.7 (SD = 0.85) at Year 10 (p = 0.003, Wilcoxon signed-rank test). The mean z-scores of the World List Generation with phonemic verbal fluency (letter P) significantly decreased from -0.60 (SD = 0.9) at Year 1 to -1 (SD = 0.9) at Year 10 (p = 0.004, Wilcoxon signed-rank test). The mean z-scores of the World List Generation with semantic (animals) verbal fluency significantly decreased from -0.5 (SD = 0.7) at Year 1 to -1 (SD = 0.9) at Year 10 (p<0.002, Wilcoxon signed-rank test). The mean z-scores of the Naming task with interference of the Stroop test did not significantly change between Year 1 and Year 10 (-0.7 (SD = 1.9) at Year 1 and -0.15 (SD = 1.3) at Year 10 (p = 0.92, Wilcoxon signed-rank test)). The mean z-scores of the Backward block spans did not significantly change between Year 1 to Year 10 (-0.4 (SD = 0.6) at Year 1 and -0.35 (SD = 0.53) at Year 10 (p = 0.45, Wilcoxon signed-rank test)).

### Relationships between global T2 lesion load changes, EDSS changes and executive functions decline during the follow-up

We found no significant correlation between T2 lesion load and EDSS changes during the follow-up (p = 0.2, Spearman Rank correlation test). We found no significant correlation between T2LL and executive functions’ changes during the follow-up (p = 0.6, Spearman Rank correlation test).

### Relationships between brain regional lesions accumulation, EDSS changes and executive functions decline during the follow-up

#### EDSS

**Fig 2:**
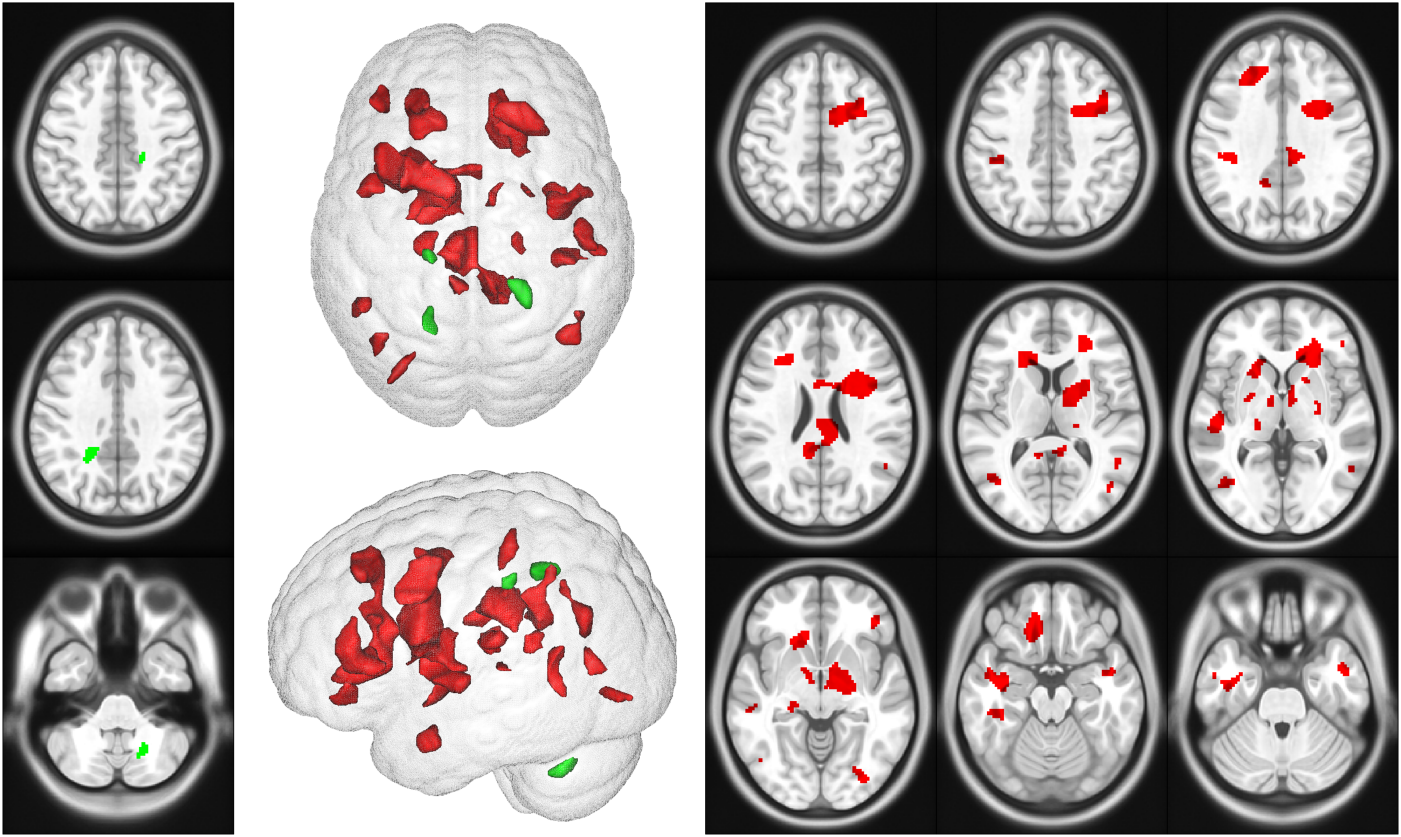
Regions where occurrence of new T2 lesions is associated with EDSS worsening (green) or decline of cognitive functions (red) during the follow-up (p<0.05, k = 20, FWE corrected at voxel level).

EDSS worsening during the follow-up was significantly associated with new or enlarged T2 lesions located in the left cerebellum, and both semi ovale centers (p<0.05, k = 20, FWE corrected) (Fig 2, Table 2).

**Table 2:** Talairach’s coordinates of the regions where occurrence of new T2 lesions is associated with EDSS progression during the follow-up.

x	y	z	p	t	Location
-18	-58	-37	0.008	6.2	Left cerebellar posterior lobe
-20	-27	40	0.018	5.78	Right semi ovale center
22	-43	32	0.022	5.69	Left semi ovale center

#### Executive functions

Fig 2 and Table 3 report the clusters where the occurrence or enlargement of T2 lesions between Year 1 and Year 10 was associated with executive functions decline (p<0.05, k = 20, FWE corrected).

**Table 3:** Talairach’s coordinates of the regions where occurrence of new T2 lesions is associated with decline of executive functions during the follow-up. (WM: white matter).

x	y	z	p	t	Z	K	location
-30	10	49	0.001	7.40	5.29	2223	WM of the left frontal lobe
-24	7	25	0.001	7.28	5.24	2223	WM of the left frontal lobe
-32	4	49	0.001	7.07	5.15	2223	WM of the left frontal lobe
-36	8	42	0.001	7.07	5.15	2223	WM of the left middle frontal gyrus
-44	15	36	0.003	6.64	4.96	2223	WM of the left frontal inferior gyrus
-20	8	42	0.013	5.93	4.61	2223	WM of the left frontal lobe
-28	-11	4	0.035	5.45	4.35	2223	Left Lentiform nucleus
-20	-12	-4	0.035	5.45	4.35	2223	Left Lentiform nucleus
-20	10	1	0.045	5.33	4.29	2223	Left Lentiform nucleus
-8	-2	-2	0.045	5.32	4.28	2223	Fornix
-8	4	-4	0.045	5.32	4.28	2223	Deep WM of the left hemisphere
-6	0	2	0.045	5.32	4.28	2223	Deep WM of the left hemisphere
-14	2	-7	0.046	5.32	4.28	2223	WM of the left frontal lobe
-26	39	10	0.001	7.27	5.24	319	WM of the left frontal lobe
44	-66	9	0.008	6.19	4.74	144	WM of the right temporal lobe
38	-4	-32	0.013	5.93	4.61	281	WM of the right temporal lobe
52	-1	-20	0.017	5.83	4.55	281	WM of the right temporal lobe
35	-9	-25	0.023	5.66	4.47	281	WM of the right temporal lobe
26	28	25	0.014	5.91	4.6	932	WM of the right frontal lobe
20	34	17	0.014	5.89	4.59	932	WM of the right frontal lobe
16	26	-17	0.03	5.53	4.40	932	WM of the right frontal lobe
22	28	8	0.035	5.44	4.35	932	WM of the right frontal lobe
14	-44	17	0.025	5.62	4.44	588	Corpus callosum
8	-40	24	0.029	5.62	4.41	588	Posterior cingulum
-34	-80	1	0.019	5.76	4.52	50	WM of the left occipital lobe
-18	-25	14	0.020	5.73	4.50	87	Deep WM of the left hemisphere
38	-27	36	0.021	5.70	4.49	122	WM of the right frontal lobe
-40	42	-10	0.028	5.56	4.41	71	WM of the left frontal lobe
-8	-42	8	0.029	5.55	4.41	53	Posterior Cingulum
55	-29	-5	0.012	5.54	4.40	38	WM of the left temporal lobe
47	-21	4	0.004	5.52	4.39	90	WM of the left temporal lobe
-44	-69	14	0.009	5.51	4.38	51	WM of the left temporal lobe
40	-34	-15	0.019	5.49	4.38	22	WM of the right temporal lobe
20	-27	-2	0.012	5.49	4.37	39	Midbrain
26	-2	2	0.02	5.44	4.35	20	Right putamen
8	-6	0	0.011	5.40	4.32	42	Deep WM of the right hemisphere
-46	1	-22	0.004	5.38	4.32	89	WM of the left temporal lobe
-52	-53	27	0.004	5.38	4.30	89	WM of the left temporal lobe
-50	-51	21	0.005	5.34	4.29	75	WM of the left temporal lobe

## Discussion

The present study provides two main findings: i) frequency and severity of cognitive impairment increase during the first ten years of RRMS; ii) clinically silent T2 lesions significantly underline cognitive decline.

### Evolution of cognitive impairment during the first 10 years of RRMS

In the present study, in order to confidently characterize the cognitive deficits of MS patients, we performed an extensive neuropsychological battery including 22 measures exploring the main cognitive functions known to be affected in MS (memory, attention/speed of information processing and executive functions). A function was considered to be affected only when the mean Z-scores of all tests exploring this function was below 1.5 [9]. This approach limits the probability that failure of one single task may be interpreted as a deficit of the entire cognitive function. This method allowed demonstrating that cognitive impairment is frequent from the first stage of RRMS and significantly increases during the first decade of the disease. Existence of cognitive impairment in patients at the first stage of the disease has been previously demonstrated [7–12]. These studies mainly evidenced attention and speed of information processing dysfunction in early MS patients. The present study confirms these findings evidencing that attention and speed of information processing deficiencies are the most frequent cognitive impairment at the first stage of the disease affecting about one third of the patients. Moreover, the longitudinal design of the present study enabled to demonstrate that frequency and severity of cognitive impairment increases during the first ten years of MS. In particular, we evidenced that patients with severe cognitive deficit (at least two domains impaired) reached from 19% at Year 1 to 39% at Year 10. These results are in accordance with the study of Amato and colleagues [21] demonstrating that frequency of cognitive impairment was 26% in patients included during the first three years of MS and reached 56% ten years later.

During the following period, frequency of attention/speed of information processing deficits only slightly increased (27% of patients at Year 1 and 38% at Year 10, the difference did not reach significance). In contrast, while executive dysfunction is rare at the first stage of the disease (only 11.5% of the patients), it increases dramatically during the follow-up, affecting 42% of patients at Year 10. This is in line with numerous studies evidencing executive dysfunction in MS. During the ten-year period, the performances of four out of the six tasks exploring the executive functions showed significant decrease. These tasks that include the TMTB, the Backward digit span and the verbal fluencies explore different aspects of the executive functions suggesting an impact of MS pathology on different subsystems of executive functions. This is in line with numerous studies evidencing executive dysfunction in MS [22–24]. Recently, Cerezo Garcia and colleagues have performed an extensive neuropsychological battery exploring executive functions and have demonstrated that alteration of these functions is frequent in MS and mainly affect three components namely, cognitive flexibility, inhibition and abstraction ability [25].

The evolution pattern of cognitive decline depicted in the present study, suggests that cognitive impairment in RRMS is characterized by early attention/speed of information processing deficiencies and subsequent executive dysfunctions. These cognitive functions may be highly dependent to the efficiency of long distant brain connectivity that may be significantly affected by white matter pathology occurring from the first stage of the disease [26]. In that way, Deloire and colleagues evidenced that the existence of diffuse brain damage in the first four years of the disease was the best predictor of subsequent cognitive deterioration seven years later [27].

#### Association between regional brain T2 lesions accrual, cognitive decline and EDSS progression during the first 10 years of RRMS

The present study evidenced that cognitive decline during the first ten years of RRMS is highly associated with the occurrence of new brain T2 lesions in the frontal, temporal and parietal lobes. The impact of brain WM lesions on cognitive functions has been previously reported in numerous cross-sectional studies performed in RRMS [28–36]. Recently, Papadopoulou and colleagues provided important findings demonstrating that WM demyelinating lesions better explain cognitive deficits than cortical lesions or cortical atrophy [37]. The present results provide another clue evidencing that among all brain lesions, those occurring in the frontal, parietal and temporal lobes were associated with cognitive decline. These findings corroborate the results obtained in previous cross-sectional studies demonstrating the particular involvement of frontal and parietal lesions on cognitive deficits [4, 38]. In addition, we demonstrated here, using extensive neuropsychological battery, that the evolution of cognitive impairment during the first ten years of MS is mainly characterized by decline of executive functions. It makes sense that MS lesions accumulation in the frontal, parietal and temporal lobes alter executive functions underlined by distributed brain neuronal networks involving mainly the frontal but also the temporal and parietal lobes. Cognitive impairment in MS may be underlined by disconnection of several brain networks secondary to macroscopic and microscopic lesions [39–41].

Most importantly, the present MRI findings argue for the differential impact of brain lesions accumulation on cognitive versus physical disability worsening. While lesions accumulation in about any regions of the frontal, parietal and temporal lobes during the first ten years of RRMS is associated with cognitive decline, only lesions accumulation in more restricted areas, namely the left cerebellum and the semi-ovale centers, impact physical disability progression. Involvement of these two last regions in motor functions probably explains the associations with EDSS that mainly traduces motor deficits. These results are in line with MRI studies performed in early RRMS patients demonstrating that cognitive deficits may be the clinical expression of early diffuse brain injury [27, 42]. Cognitive deficits may be underlined by the macroscopic lesions occurring in various brain regions combined with microscopic damage of the normal appearing brain tissue [43]. All these findings suggest that cognitive decline is probably a sensitive marker of more severe diffuse brain disease characterized by macroscopic and microscopic demyelinating lesions.

The present study suffers from several limitations. First, the sample size is small and it would be necessary to validate the present results in a second larger cohort. Secondly, we cannot exclude a residual test re-test effect while the exclusion of the baseline cognitive data has probably limited this effect. This may partly explain that the proportion of patients with abnormal cognitive tests remains limited at year 10. Finally, the MR scanner has been changed between year 1 and year 10 preventing any confident assessment of brain atrophy.

## Conclusion

During the first ten years of MS, cognitive impairment increases and is mainly characterized by early deficit in attention/speed of information processing and subsequent decline of executive functions. MRI follow-up demonstrates that brain T2 lesions accumulation mostly impact cognition rather than physical performances. In daily practice, early prevention of brain T2 lesions accrual may be useful to limit cognitive decline.
